# In Situ Electric‐Induced Switchable Transparency and Wettability on Laser‐Ablated Bioinspired Paraffin‐Impregnated Slippery Surfaces

**DOI:** 10.1002/advs.202100701

**Published:** 2021-05-29

**Authors:** Chao Chen, Zhouchen Huang, Suwan Zhu, Bingrui Liu, Jiawen Li, Yanlei Hu, Dong Wu, Jiaru Chu

**Affiliations:** ^1^ CAS Key Laboratory of Mechanical Behavior and Design of Materials Department of Precision Machinery and Precision Instrumentation University of Science and Technology of China Hefei 230026 China

**Keywords:** bioinspired electric‐triggered slippery surfaces, femtosecond lasers, in situ controllable wettability, switchable visibility, transparent silver nanowire thin‐film heater

## Abstract

Switchable wetting and optical properties on a surface is synergistically realized by mechanical or temperature stimulus. Unfortunately, in situ controllable wettability together with programmable transparency on 2D/3D surfaces is rarely explored. Herein, Joule‐heat‐responsive paraffin‐impregnated slippery surface (JR‐PISS) is reported by the incorporation of lubricant paraffin, superhydrophobic micropillar‐arrayed elastomeric membrane, and embedded transparent silver nanowire thin‐film heater. Owing to its good flexibility, in situ controllable locomotion for diverse liquids on planar/curved JR‐PISS is unfolded by alternately applying/discharging low electric‐trigger of 6 V. Simultaneously, optical visibility can be reversibly converted between opaque and transparent modes. The switching principle is that in the presence of Joule‐heat, solid paraffin would be melt and swell within 20 s to enable a slippery surface for decreasing light scattering and frictional force derived from contact angle hysteresis (*F*_CAH_). Once Joule‐heat is discharged, undulating rough surface would reconfigure by cold‐shrinkage of paraffin within 8 s to render light blockage and high *F*_CAH_. Upon its portable merit, in situ thermal management, programmable visibility, as well as steering functionalized droplets by electric‐activated JR‐PISSs are successfully deployed. Compared with previous Nepenthes‐inspired slippery surfaces, the current JR‐PISS is more competent for in situ harnessing optical and wetting properties on‐demand.

## Graphical abstract

Switchable optical and wetting properties are highly desirable for up‐to‐date smart surfaces. By embedding a portable, low‐voltage‐driven and transparent silver nanowires heater, the sandwich‐structured bioinspired Joule‐heat‐responsive paraffin‐impregnated slippery surface (JR‐PISS) is competent for in situ tuning these two features in synergy. Insights into the rational design of electric‐induced actuator offer a platform for developing and optimizing next‐generation smart windows.

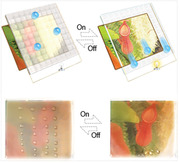

## 1 Introduction

In the past decade, smart surfaces with reversibly switchable wettability have attracted pronounced attentions on account of their great potential in both scientific investigations and industrial applications, involving antifouling,^[^ ^1^ ^]^ droplet‐based microfluidics,^[^ ^2^ ^]^ oil–water separation,^[^ ^3^ ^]^ “no‐loss” droplet transportation,^[^ ^4^ ^]^ cell adhesion,^[^ ^5^ ^]^ anti‐icing,^[^ ^6^ ^]^ biotechnology,^[^ ^7^ ^]^ and so forth. Accordingly, through artificially fabricating fine‐tuned micro‐structured topography and developing stimuli‐responsive materials, people have tactfully designed a wide variety of smart surfaces responsiveness to external stimulus such as electric‐field,^[^ ^8^ ^]^ photo‐irradiation,^[^ ^9^ ^]^ magnetism,^[^ ^10^ ^]^ temperature,^[^ ^11^ ^]^ pH,^[^ ^12^ ^]^ or mechanical strain,^[^ ^13^ ^]^ aiming at realizing the dynamically switching wettability of their surface liquids. For example, Jiang and his coworkers conferred a kind of conductive lubricant‐infused porous poly(3‐hexylthiophene) fiber by utilizing a one‐step freeze‐drying method, which is competent for the reversible control of a drop's locomotion via switching between the Cassie state (slippery state) and the transition state between the Cassie state and Wenzel state (non‐slippery state) by alternately removing and applying electric stimuli.^[^ ^14^ ^]^ Gao et al. developed a photo‐responsive organogel surface for realizing the controllable sliding velocity and intentional slipping route of water drops by integrating the photothermal effect of Fe_3_O_4_ nanoparticles and the low hysteresis characteristic of lubricant‐infused organogels.^[^ ^9c^ ^]^ The underlying mechanism is assigned to asymmetric Laplace force acting on the meniscus derived from the lubricant temperature‐gradient‐induced surface tension difference, that is, wettability gradient force. More recently, Guo et al. reported a sort of amphibious slippery surface by combining magnetically responsive iron powder doped elastomer and silicone oil, which is adaptive for manipulating the wettability of water drops and underwater bubbles depending on the magnetic‐controlled topography.^[^ ^15^ ^]^ Unfortunately, all these electric/light/temperature/magnetism‐triggered surfaces are only serving for one single functionality but hard to satisfy our up‐to‐date requirements.

Apart from controllable wettability, smart surfaces that have a synergistic switchable optical transparency may open opportunities for multifunctional application, such as tents that block light on a dry sunny day, but become both transparent and water‐repellent on a dim rainy day; adaptive anti‐ice surfaces that adjust from droplet‐impact‐resistant topographies in cold, dry weather, to a condensation‐preventive smooth surface in humid conditions; and more. With this in mind, Aizenberg's group unfolds the first paradigm of adaptive fluid‐infused porous films with switchable optical transparency and wettability that respond to a graded mechanical stimulus.^[^ ^16^ ^]^ By alternately loading and discharging the mechanical stretch, the surface topography can be readily switched between an undulating rough morphology and a homogeneous flat one, thereby immobilizing/slipping the surface water droplets and synergistically reflecting/transmitting the incident light on this slippery liquid‐infused porous surface (SLIPS). Afterward, by doping the thermo‐responsive phase change material of n‐paraffin into the organogel, Yao et al. prepared a temperature‐sensitive smart surface for regulating both the optical transmittance and its surface adhesion force.^[^ ^17^ ^]^ Once the surface temperature is elevated above the melting point of n‐paraffin, the melt paraffin donates a liquid lubricant layer for decreasing the surface roughness to slip a surface droplet and simultaneously enhancing the transmittance of incident light. On this basis, Manabe et al. presented a temperature‐activated paraffin‐infused porous surface by using a layer‐by‐layer self‐assembly method, where the optical property and surface wettability dramatically exhibit the room‐temperature‐reliant variation via optimizing the hybrid ratio of solidifiable/liquid paraffin.^[^ ^18^ ^]^ Though ceaseless efforts have been dedicated to advancing the experimental and theoretical understanding of SLIPS‐based smart surfaces (Table S1, Supporting Information), several blockages arise subsequently: 1) The water‐proof porous platform is typically fabricated by utilizing chemical strategy, which is not environment‐benign but time‐consuming.^[^ ^18^ ^]^ 2) Switching strategy is limited to an ex situ hot plate or oven with an exaggerated voltage of 220 V, which is not portable but energy‐consuming.^[^ ^17^ ^]^ For mechanical method, the repeating stretching stress is prone to result in a short longevity over mechanic‐triggered actuator and the volatile lubricant (perfluoropolyether) further impedes its long‐term usage in tuning optical and wetting properties.^[^ ^16^ ^]^ For the typically self‐adaptive hydrogel or organogel materials, the conversion of optical and wetting properties is room‐temperature‐reliable, which may not work in some harsh ambient when the outside temperature is always subzero in winter and thus this passive tuning strategy is disabled.^[^ ^11^, ^18^ ^]^ 3) Absence of dynamic spectrum analysis results in an elusive thermal‐response process (e.g., response time), which further hinders the deeper understanding over the switching mechanism.^[^ ^16^, ^17^, ^18^ ^]^ 4) To the best of our knowledge, in situ encoded visibility and tunable wettability on flexible smart surfaces have been rarely demonstrated. As a result, seeking a more facile, ecofriendly, energy‐saving and portable smart surface for realizing in situ programmable optical transparency and controllable wettability, as well as uncovering the underlying thermodynamics, is still a timely challenge and an urgent need.

**Figure 1:**
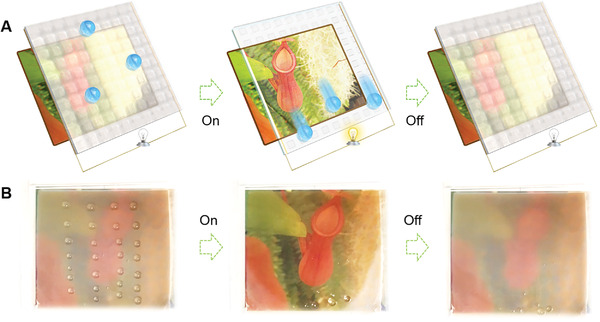
Conceptual bioinspired intelligent window with switchable optical visibility and surface wettability. A) Schematic diagram of an electric‐triggered device inspired by natural *Nepenthes* picher plant for tuning surface optical and wetting properties. Once its surface is impregnated, paraffin wax is solidified without Joule‐heat, liquids tend to stick and appearance is opaque. In sharp contrary, once the impregnated paraffin wax is liquefied upon Joule‐heat, liquids would slide and appearance is transparent. B) a proof‐of‐concept of regulating optical transparency and wettability simultaneously on an ultra‐low voltage‐actuated electronic (area: 6×6 cm^2^) by remotely loading and discharging Joule heat (serving voltage: 10 V). The result unfolds that current newly explored electronic is competent for in‐situ steering optical and wetting properties in synergy, and has an excellent self‐cleaning ability.

In this work, we report a sandwich‐structured Joule‐heat‐responsive paraffin‐infused slippery surface (JR‐PISS) by the integration of thermo‐activated phase‐change‐material of paraffin, superhydrophobic micropillar‐arrayed elastomeric membrane (SPAM) fabricated by one‐step femtosecond laser ablation, and an embedded transparent silver nanowire thin‐film heater (SNWH). Taking the advantage of its good flexibility, in situ controllable wettability and locomotion for diverse liquids on 2D/3D JR‐PISS is realized through alternately loading/discharging a low voltage of 6 V. Synergistically, light transmission can be reversibly switched between opaque and transparent modes in response to alternate electric‐trigger (**Figure** **1**; Movie S1, Supporting Information). The switching principle is that the slippery liquefied‐paraffin layer enables JR‐PISS an enhanced optical transparency and low hysteresis when Joule‐heat is applied to elevate the surface temperature above the melting point of paraffin wax (*T*_m_) within 20 s. Upon removing Joule‐heat, undulating non‐slippery JR‐PISS would reconfigure by the cold‐shrinkage of solidified paraffin within 8 s to obstruct the transmittance of incident light and increase the surface energy barrier. Moreover, the quantitative relationship among serving voltage and surface temperature and response time, together with the on–off state transparency as a function of laser power, is systematically investigated. Owing to its broadband switchable light range, a derived home‐made optical tunable window for thermal management is successfully deployed. Significantly, thanks to its portable merit, in situ programmable visibility by selectively switching isolated JR‐PISSs is presented. Compared with the previously reported Nepenthes‐inspired smart surfaces, this versatile JR‐PISS is more competent for simultaneously harnessing light transparency and wettability according to users’ request. More significantly, compared to other bi‐functional SLIPS, current JR‐PISS is more convenient, integrated, flexible, portable, and energy‐saving. This study provides insights for dealing with the challenges of bi‐functional thermo‐involved smart surfaces.

## 2 Results and Discussion

### 2.1 Facile Fabrication of Bi‐Functional JR‐PISS

**Figure 2:**
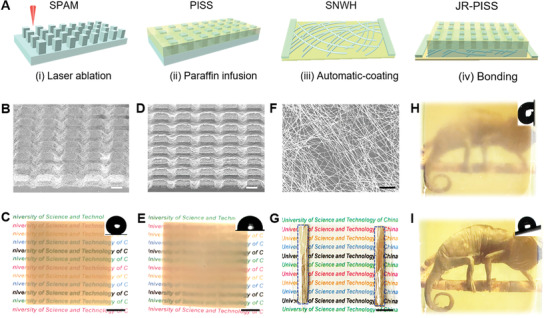
Fabrication of bi‐functional JR‐PISS by combining PISS and transparent SNWH. A) Schematic strategy for preparing electric‐triggered JR‐PISS including manufacturing super‐hydrophobic SPAM, impregnating lubricant paraffin into SPAM by thermal‐spin‐coating, weaving SNWH using automatic roll‐to‐roll method and integrating the resultant PISS together with transparent SNWH. SEM images for laser‐ablated SPAM B) without and D) with paraffin wax; The scale bar is 50 *μ*m. Digital pictures for laser‐ablated SPAM C) without and E) with paraffin wax; The scale bar is 1 cm and the insets are the characterized WCA on their corresponding surfaces. F) SEM clip of the as‐prepared silver nanowires; The scale bar is 2 *μ*m. G) Photograph for silver nanowires‐woven heater; The scale bar is 1 cm. Visibility switching by alternately H) ldischarging and I) loading electric‐stimuli of 6 V; The insets represent the corresponding SA conversion in response to alternate electric‐trigger.

Fabricated is a sandwich‐structured JR‐PISS having three key components, including the topside lubricated paraffin wax, middle SPAM, and an underlying transparent SNWH. Accordingly, its manufacturing protocol contains femtosecond laser ablation, paraffin impregnation, silver‐nanowire‐coating, and SNWH bonding, where the cost is roughly estimated as low as ≈3.65 $ (**Figure** **2A** and Table S2, Supporting Information).

SPAM displays three crucial roles in determining the performance of JR‐PISS: 1) Conferring a giant capillary force for the lubricant adsorption, spreading, and storage enables an excellent durability over JR‐PISS. 2) Providing a robust resistance for obstructing the intrusion of objective liquids into JR‐PISS. 3) Donating a low reflection coefficient, which is a prerequisite for switching the optical property of JR‐PISS. In this regard, a piece of SPAM, that is superhydrophobic micropillar‐arrayed polydimethylsiloxane membrane with a static water contact angle (WCA) of ≈151^o^, can be readily fabricated by one‐step femtosecond laser cross‐scanning (Figure 2B,C). Therein, the typical width (*w*), height (*h*), and interval (*d*) for uniformly arrayed micro‐pillars were characterized as 50.0, 46.0, and 100.0 *μ*m (Figures S1 and S12f, Supporting Information), respectively. Given a further investigation of its surface morphology, the resultant SPAM is dramatically accompanied by numerous nanostructures, which is conducive to providing a large capillary force acting on the targeted lubricant. In comparison with traditional chemical hydrophobized methods,^[^ ^18^ ^]^ current one‐step fs laser cross‐scanning is more facile, tunable, efficient, and ecofriendly on account of its advantages of high fabrication efficiency and accuracy, which is thus widely adaptive for modifying the surface wettability with respect to various materials.^[^ ^19^ ^]^

Paraffin, behaving as a lubricant in response to thermo‐stimulus, is responsible for switching the surface roughness and visibility via a reversible solidified‐liquefied transformation. Selected is paraffin wax with a *T*_m_ of 52 °C in consideration of its merits of low‐toxicity, non‐volatility, and good biocompatibility. By means of thermal‐spin‐coating method, the solid paraffin wax could be homogeneously infused into the as‐prepared SPAM for a derived paraffin‐infused slippery surface (PISS) after a brief thermal‐spreading and cold‐shrinkage process, where the apparent WCA of PISS is characterized as ≈94^o^ (Figure 2D,E and Figures S2 and S3, Supporting Information). Therein, the thickness of paraffin layer could be roughly measured as 29.1(±2.3) *μ*m upon subtracting the 3D profile of SPAM from PISS (Figure S14d,g, Supporting Information). Additionally, compared with SPAM, the optical property for PISS is further decreased because of the increased light scattering/reflectance arising from the uneven solidified paraffin layer. Notably, lubricated paraffin layer behaves not only as a transparent–opaque switch but also a slippery–nonslippery converting dominator, which is therefore another critical component accountable for the dynamic glazing of JR‐PISS.

Underlying transparent heater, enabling JR‐PISS an admirable self‐heated capability without the assistance of ex situ facility (e.g., hot plate or oven), is functional for in situ harnessing the surface paraffin switching between liquefied and solidified states. A desirable heater is supposed to satisfy four following criterion: a superior optical transparency, good portability and flexibility, low energy‐cost, and fast thermal‐response. Fortunately, silver nanowire thin‐film heater (SNWH) deserves all these standards because of its virtues of ultrahigh figure‐of‐merit, ultralow haze, durable mechanical compliance, together with excellent electric and thermal conductivity.^[^ ^20^ ^]^ Notably, we employ the heater woven by silver nanowires rather than those by carbon nanotubes or graphene, just because the former has a lower conjunction‐resistance (≈2 kΩ) than that of latter (≈0.3–4 MΩ),^[21]^ indicating that carbon materials have higher driven voltages and thus exaggerated energy consumption. Accordingly, the highly purified silver nanowires (SNWs) with aspect‐ratio above 1000 could be harvested by a classical polyol method,^[^ ^20a^ ^]^ where the statistic length and diameter for purified SNWs are 21.5(±2.5) *μ*m and 21.0(±1.5) nm (Figure 2F and Figure S4, Supporting Information), respectively. Thereafter, the rheology‐modified SNW ink (1 mg mL^−1^) could be automatically coated on polyethylene terephthalate (PET) substrate for a SNW‐woven transparent conductive thin‐film with an optical transmittance of 90.0% and sheet resistance of 97.2(±8.0) Ω sq^−1^ (Figure S5, Supporting Information). Afterward, a robust transparent SNWH could be harvested by successive symmetric electrode molding using silver paste, copper wire welding, and a subsequent annealing treatment (Figure 2G). Finally, a fresh JR‐PISS integrated by PISS and SNWH could be successfully fabricated through chemical bonding depending on a thin layer of cured adhesive PDMS, which is capable of steering the dynamical switching of surface optical property between opaque and transparent states by remotely loading/discharging a low direct current (DC) voltage of 6 V (Figure 2H,I; Movie S2, Supporting Information). Simultaneously, the surface wettability could be regulated between nonslippery state (pinning) and slippery one (sliding angle ≈24^o^) (Figure S6, Supporting Information). Moreover, the longevity tests verify that this electric‐triggered actuator unfolds good stability in terms of long‐term serving, bending, twisting, and heating–freezing for steering the wetting and optical properties (Figures S7 and S8, Supporting Information). Significantly, in comparison with previously reported smart surfaces,^[^ ^16^, ^17^, ^18^ ^]^ this scalable, flexible, portable, and energy‐saving JR‐PISS is more competent for reversible in situ regulating light and wetting properties by taking advantage of its good reconfiguration capability, which is thus conducive for newly explored smart windows due to its self‐cleaning feature.

### 2.2 Switching Principle for Electric‐Triggered JR‐PISS

**Figure 3:**
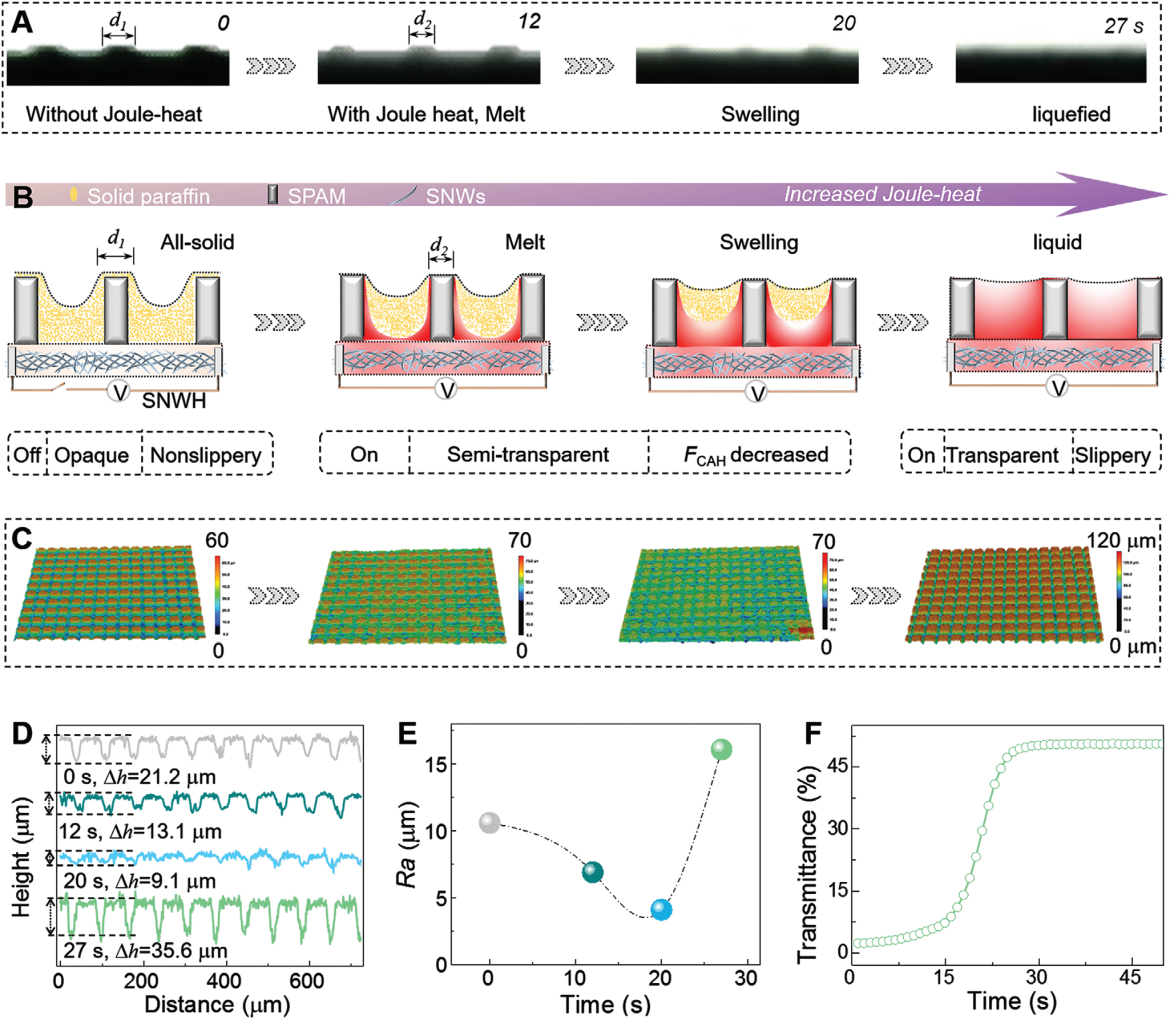
Switching principle enabled by in situ monitoring the surface topography evolution of JR‐PISS. A) Sectional profile variation in terms of Joule‐heating time. B) Schematic model for surface morphology evolution suggested by (A). C) Top profile detection, D) line‐scanning curves, and E) surface *R_a_* change with Joule‐heating time. F) Optical transparency variation as the function of Joule‐heating time. The results uncover that the surface suffering from melt, swelling and a subsequent liquefied process donates the switch between reflective/scattered nonslippery topography and slippery one.

By taking advantage of the portable merit of JR‐PISS, the topography evolution of surface lubricant in terms of Joule‐heating time could be in situ monitored by using a camera equipped with high‐resolution CCD (**Figure** **3A**; Movie S3, Supporting Information). In sectional view, the observed dynamic procedure in the presence of Joule‐heat is composed of four following clips (Figure 3B): i) voltage on, *T* < *T*_m_. The concave solidified‐paraffin layer is prone to retain its original all‐solid state for a rough undulant appearance. ii) Until the Joule‐heating time comes to 12 s, the underlying solid paraffin at pillar‐ and root‐adjacent domains start to melt preferentially since their localized temperature has been elevated above *T*_m_ (Figure S9, Supporting Information). iii) With the increased thermal input, the underlying increased liquefied‐paraffin elevates the lubricant layer upward by virtue of swelling. iv) Finally, the semi‐solid paraffin could be entirely liquefied to submerge SPAM for a smooth terrain. This dynamic morphology‐evolving could be co‐operatively verified through in situ observation of JR‐PISS from side view by utilizing an optical microscope, where the surface lubricant reversibly switching between solidified feature and liquefied one was further manifested (Figure S10 and Movie S4, Supporting Information).

To further confirm the above suggested model, we employ a 3D optical profile meter to investigate the surface topography transformation as a function of the Joule‐heating time (Figure 3C,D). 3D clips threw a visually rational case that the detectable solid paraffin layer was gradually elevated toward plateau by the underlying preferentially liquefied‐paraffin with the increasing of Joule‐heating duration. The corresponding *R*_a_ value dynamically decreased from 10.6 to 6.9, 4.1 *μ*m, and then increased to 16.1 *μ*m, implying that the light transmittance of JR‐PISS in response to Joule‐heating involved a roughness‐dominated process derived from swelling and a subsequent transparent one derived from liquefied‐paraffin (Figure 3E). Furthermore, we harnessed dynamic spectrum analysis for in situ detecting the optical property change of JR‐PISS with Joule‐heating time, where we found that the optical transmittance increased exponentially from 2.1% to 5.0%, 23.0% and then 49.3% (Figure 3F). On this basis, the controllable wettability and switchable transparency could be readily realized by virtue of in situ loading or discharging electric‐stimuli for a slippery air/liquefied‐paraffin/solid‐SPAM (ALS) system or a rough nonslippery air/solidified paraffin/solid SPAM (ASS) one. In brief, comprehensive analysis by topography and optical detecting techniques enables us a deeper understanding of switching principle over electric‐activated JR‐PISS, which is envisioned to provide a profound guidance for researchers occupied in light‐ and wetting‐involved smart surfaces.

### 2.3 Thermodynamics over Transparency Switching

Regardless of sheet resistance (*R*), the serving voltage (*U*) applied on SNWH is therefore regarded as the dominator of Joule‐heat output (*Q*) according to classical Joule's law:

(1) $$Q=\frac{U^{2}}{R}t$$

**Figure 4:**
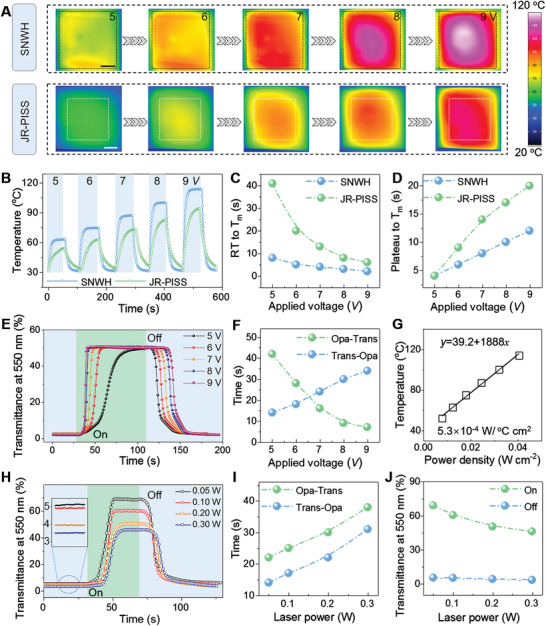
Clarification of thermo‐dynamics for in situ switchable transparency by dynamic‐spectrum‐analysis. Comparisons of A) thermal infrared images, B) temperature–time curves, C) time‐spent for rising above *T*_m_ (52^o^), and D) time‐consumed for decaying below *T*_m_ over embedded SNWH and JR‐PISS under serial electric‐triggers; the scale bar is 1 cm. The larger the applied voltage, the more the generated Joule‐heat. E) Optical transparency as a function of time curves and F) Durations of opaque‐transparent (3.8–50%) and transparent–opaque (50–10%) under serial electric‐stimulus. The larger the applied electric‐trigger, the faster the velocity of reaching plateau. G) Temperature variations in terms of power density and their linear‐fitting curve for calculating the energy consumption assigned to current SNWH. H) Transparency‐time curves and I) switching durations of opaque‐transparent and transparent‐opaque (plateau‐10%) and J) on–off transparency comparison for JR‐PISSs resultant from laser powers of 0.05, 0.1, 0.2, and 0.3 W, where the applied voltage is fixed as a constant of 6 V. The larger the laser power, the smaller the on–off transparency.

Accordingly, we study the influence of *U* on the surface temperature and thermal‐response time assigned to SNWH and JR‐PISS for quantitatively uncovering the heat‐transfer process among medium (**Figure** **4A**). The obtained thermal infrared images suggested that the Joule‐heat is prone to increase with the elevation of *U*. For clarity, the temperature evolution depending on time curves for confirming the plateau of SNWH and JR‐PISS was then implemented (Figure 4B). With the increase of *U* from 5 to 6, 7, 8, and then 9 V, the plateau for underlying SNWH elevated from 62.6 to 74.3, 86.9, 100.0, and then 113.6 °C, and the peak for JR‐PISS climbed from 53.8 to 62.6, 73.2, 82.7, and then 94.2 ^o^C, respectively. The larger the applied serving voltage, the more the generated Joule heat. Accordingly, under serial electric‐stimulus, the time spent on elevating the surface temperature above *T*_m_ for SNWH was recorded as 8, 5, 4, 3, and 2 s in comparison with that for JR‐PISS as 41, 20, 13, 8, and 6 s (Figure 4C), respectively. The larger the applied electric‐trigger, the faster the velocity of reaching targeted *T*_m_, and the slower the velocity of decaying below *T*_m_ (Figure 3D). Notably, the slower rising/decaying velocity of JR‐PISS compared with SNWH is supposed to be determined by a time constant (*τ*) defined as^[^ ^22^ ^]^

(2) $$\tau =\frac{\rho dc}{h}$$

where *ρ*, *d*, *c*, and *h* denote the mass density, thickness, specific heat capacity, and heat‐transfer coefficient, respectively. These parameter values for various materials could be seen from Table S3, Supporting Information. Therein, paraffin wax lubricated in JR‐PISS having a far smaller *h* value (0.12 W m^−1^·K^−1^) than that of the underlying SNWs (429 W m^−1^·K^−1^) should be responsible for its slower heat‐transfer from embedded SNWH to PDMS, paraffin wax, and then air. Besides, the paraffin wax having a larger *c* value (2.6 J/g °C) leads to a slower decaying velocity of JR‐PISS. Considering that *τ* is linearly proportional to *d*, researchers can further optimize the performance of JR‐PISS with regard to the thermal response time by virtue of reducing the thickness of PDMS and paraffin layers in a future work.

Based on above‐explored thermodynamics, the switching speed for opaque‐transparent and transparent‐opaque transformation by using JR‐PISS under serial electric inputs could be unfolded by employing optical dynamic‐spectrum‐analysis (Figure 4E). Under the applied voltages of 5, 6, 7, 8, and 9 V, the opaque‐transparent transformation for JR‐PISS endured 42, 28, 16, 9, and 7 s and the transparent‐opaque conversion time was obtained as 14, 18, 24, 30, and 34 s (Figure 4E), respectively. The trend is highly consistent with above explored thermodynamics‐involved results. For practical usage, smart surfaces with low energy consumption are always highly admirable in regard of their economical merit. Herein, according to an empirical formula^[^ ^22^ ^]^

(3) $$E=\frac{U^{2}}{ART}$$

where *E*, *A*, *R*, *U*, and *T* signify the energy consumption, area, sheet resistance, applied voltage, and corresponding temperature for SNWH, respectively. We could harvest an ultralow *E* ≈ 5.3 × 10^−4^ W per °C cm^2^ by virtue of linear fitting *T* as a function of power density, which is comparatively superior to a wide variety of reported heaters (Figure 4G and Figure S11, Supporting Information). That is, SNWH is more competent for engineering the thermo‐involved smart surfaces ascribed to its energy‐saving advantage.

Apart from electric input, the morphology influence of SPAM on the optical switching performance of JR‐PISS was further investigated by dynamic‐spectrum‐analysis (Figure 4H and Figure S12, Supporting Information). Therein, the morphology of micro‐pillars including their featuring width (*l*), height (*h*), and interval (*d*) are determined by the programmable laser ablation, which affects the on–off transparency of JR‐PISS through influencing the ratio of incident‐light scattering and incident‐light penetrating through (Figure S13, Supporting Information). That is, the larger the fs laser textured *h*, the longer the light scattering path in grooves and thus the lower the recorded optical transparency. As such, the larger *d* and *l* would elevate the ratio of unprocessed transparent domain relative to ablated high‐scattering one, which is therefore conducive to harvesting an optimized JR‐PISS with higher optical performance. Typically, with the increase of laser processing power, the height of micropillars (*h*) was elongated from 23.0 to 33.1, 45.8, and then to 55.2 *μ*m (Figure S14a–f, Supporting Information). Upon the identical thermal‐spin‐coating parameters, the thickness of lubricant paraffin layer for four different JR‐PISSs increased from 14.1 to 22.3, 29.1, and then to 33.0 *μ*m, resulting in a slower thermal‐response process (Figure 4I and Figure S14g, Supporting Information). Moreover, the resultant SPAMs by laser powers of 0.05, 0.1, 0.2, and 0.3 W has progressively increased surface roughness originating from enhanced laser ablation degree, which was inclined to deteriorate the optical transparency upon the increased light scattering (Figure 4J and Figure S14h, Supporting Information). Accordingly, people could on‐demand select a desirable JR‐PISS in consideration of thermal‐response time and optical transparency. In the following study, we choose a JR‐PISS fabricated by 0.20 W with an applied voltage of 6 V for controllable wettability and switchable transparency in consideration of its moderate comprehensive performance.

### 2.4 In Situ Reversibly Switchable Wettability for Diverse Liquids on 2D/3D JR‐PISS

**Figure 5:**
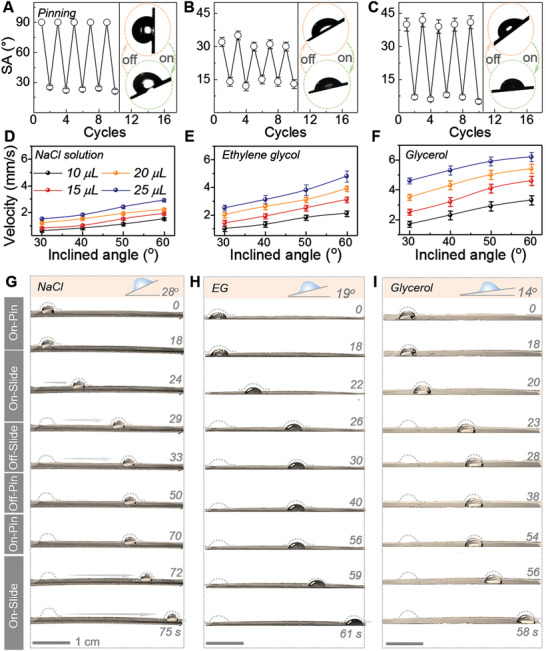
In situ reversible locomotion control over diverse liquids and their quantitative investigations. Cyclic SA variations in response to alternate electric‐trigger of 6 V for A) NaCl solution, B) ethylene glycol, and C) glycerol droplets (10 *μ*L) on a JR‐PISS. Quantitative relationship among drops volume and inclined angle and sliding velocity for droplets of D) NaCl solution, E) ethylene glycol, and F) glycerol released on an electric‐activated JR‐PISS (applied voltage: 6 V). Reversibly dynamic control between pinning and sliding by remotely applying/discharging electric‐stimuli of 6 V over diverse liquid species, including G) NaCl solution, H) ethylene glycol as well as I) Glycerol drops (10 *μ*L). Locomotion control over diverse drops by electric‐triggered JR‐PISS contains i) voltage on, *T* < *T*_m_. Stick a droplet; ii) then, the surface temperature elevates above *T*_m_ to slip a droplet; iii) voltage off, *T* > *T*_m_. The surface droplets continue to slide for a short distance until iv) the surface temperature decays below *T*_m_ to pin the targeted droplet; v,vi) display another cyclic locomotion control.

Smart surfaces that are capable of steering liquid species with diverse rheological parameters is of great importance for broadening their potential applications in anti‐fouling and anti‐icing, in addition to microfluidics. Presented is an electric‐activated JR‐PISS accountable for reversibly switching three typical liquids wettability by remotely applying/discharging electric‐trigger (**Figure** **5A**–C; Movie S5, Supporting Information). At room temperature, SAs for NaCl solution, ethylene glycol (EG), and glycerol were characterized as >90^o^ (pinning), 32 ± 3^o^, and 41 ± 2^o^, respectively. Once a voltage of 6 V was remotely loaded, the electric‐induced‐heating effect enabled JR‐PISS a slippery ALS system for decreasing their corresponding SAs to 24 ± 1^o^, 14 ± 2^o^, and 7 ± 2^o^, respectively. In addition, the quantitative relationship among drop volume and inclined angle and their sliding velocity had been examined for clarifying the hydrodynamics of these droplets on such a slippery surface (Figure 5D–F). Generally, the resistance derived from contact angle hysteresis (*F*_CAH_) could be calculated as^[^ ^23^ ^]^

(4) $$F_{CAH}=\gamma \times L\times \left(\cos\theta_{r}-\cos\theta_{a}\right)$$

where *γ*, *L*, *θ_r_*, and *θ_a_* refer to the surface tension, characteristic length, and receding and advancing angles of liquid droplet on JR‐PISS. Considering that *F*_CAH_ is proportional to *γ*, the EG droplet with a smaller *γ *(4.6 × 10^–2^ N m^−1^) having a minimum resistance should exhibit a theoretically faster sliding velocity in comparison with NaCl solution (8.5 × 10^–2^ N m^−1^) and glycerol droplets (6.2 × 10^–2^ N m^−1^). However, the experimental result unfolded that the glycerol droplet tended to move more easily, which should be ascribed to its unique moving behavior, that is, a “rolling” manner rather than a “slipping” one on such a slippery surface.^[^ ^24^ ^]^ On this basis, by slightly tilting JR‐PISS above the SAs of these three droplets, we could readily realize the locomotion control by alternately loading/discharging electric‐trigger (Figure 5G–I). Steering dynamics should be introduced as follows: i) voltage on, *T* < *T*_m_. Remote paraffin layer would maintain its original solidified morphology for a frictional ASS system so as to pin a surface droplet. ii) Until *T* > *T*_m_, the melt paraffin contributed to a slippery ALS system to slide an objective drop on this sandwich‐structured JR‐PISS. iii) Once the electric‐stimuli were intentionally switched off, the surface droplet is not capable of braking immediately on account of the redundant Joule heat. iv) After several seconds, the surface temperature decayed below *T*_m_, the solidified paraffin layer enabled a reconfigurable ASS system for sticking the targeted droplet and one cyclic locomotion control of pining‐sliding‐pinning could be successfully implemented. By the same token, another cyclic droplet locomotion control could be readily achieved seen from cases v and vi.

Significantly, by taking advantage of its good flexibility, current electric‐triggered JR‐PISS is dramatically adaptive for steering diverse functionalized droplets released on curved JR‐PISS. Figure S15a, Supporting Information displays metachromatism by actuating a blue droplet (5 *μ*L, pipetted from the as‐prepared mixture of glucose and indigo carmine) and a colorless NaOH droplet (5 *μ*L) to coalesce for a hybrid yellow one (Movie S7, Supporting Information). By the same token, two isolate colorless droplets (left one: 5 µL glycerol containing a small amount of phenolphthalein; right one: 5 µL NaOH aqueous solution) pinning on a curved nonslippery JR‐PISS can slide successively toward the bottom to aggregate into a larger pink one once the voltage is loaded for a slippery surface (Figure S15b and Movie S8, Supporting Information). Apparently, the sliding velocities for these two liquid species are very different in regard to their disparate rheological properties. For a wide usage, people can harvest various coalesce speed by simply adjusting the curvature or tilt angle of JR‐PISS on account of its robust flexibility.

### 2.5 In Situ Thermal Management and Programmable Visibility

**Figure 6:**
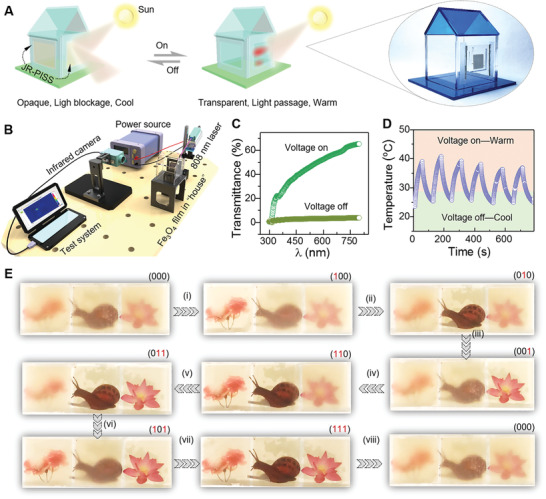
In situ thermal management, programmable optical visibility and steering diverse functionalized drops by remotely regulating electric‐actuated JR‐PISSs. A) The left unfolds a conceptual potential over thermal management as a light shutter and the right features a customized “house” equipped with JR‐PISS. B) Proof‐of‐concept system in terms of thermal management, composed of computer‐controlled program, high‐resolution infrared camera, digital power source, light source (808 nm), and Fe_3_O_4_‐doped PDMS film (5 wt%) in “house”. C) UV–Vis spectrum for recording the on–off transparency in a wide range of 300–800 nm of JR‐PISS. The curve demonstrates that current smart surface is not only adaptive for visible light but also responsive to near infrared one. D) Surface temperature regulation of indoor objective Fe_3_O_4_‐doped film by remotely switching an electric‐gate. E) Programmable visibility by intentionally switching isolate JR‐PISSs.

For practical usage, people always anticipate to in situ switch the optical transparency by using smart surfaces in an initiative manner other than an ex situ passive one. In this consideration, unfolded is a kind of remotely electric‐triggered JR‐PISS, which could be intentionally switched between transparent and opaque states upon simply switching an electrical‐gate. For example, one can regulate the incident sunlight for personal thermal management, which is therefore convinced to be favorable for enhancing the comfortable degree of human body by remotely “open” or “shut” JR‐PISS on‐demand (**Figure** **6A**). To verify this conceptual scenario, a home‐built mimic platform was successfully assembled by thermal infrared imaging test system, high‐frame‐rate infrared camera, DC power source, 808 nm light source, customized “house” equipped with JR‐PISS, and an indoor Fe_3_O_4_‐doped PDMS film (5 wt%) (Figure 6B and Figure S16, Supporting Information). Considering that JR‐PISS works in a wide range of light (400–800 nm), we could regulate the surface temperature of indoor Fe_3_O_4_‐doped membrane through allowing/blocking the incident near‐infrared light (808 nm), where the lubricant paraffin layer behaving as a light shutter is thus regarded as the dominator (Figure 6C,D; Movie S6, Supporting Information). Notably, the slow rising and decaying velocity for surface temperature of Fe_3_O_4_‐doped membrane should be dependent on the above‐explored thermo‐dynamics. In terms of personal privacy, three JR‐PISSs in parallel could be on‐demand programmed to a series of encodings ranging from all‐switched off state (000) to all‐switched on state (111) according to the user's request (Figure 6E; Movie S9, Supporting Information).

## 3 Conclusion

In summary, the reported JR‐PISS is a kind of novel intelligent electric‐triggered actuator integrated by superhydrophobic SPAM resultant from one‐step fs laser cross‐scanning, impregnated paraffin wax, and transparent SNWH, which is driven by an ultralow energy consumption of 5.3 × 10^–4^ W per °C cm^2^. Owing to its good flexibility, in situ reversibly switchable wettability for diverse liquids on 2D/3D surfaces has been realized through remotely loading/discharging a low electric‐stimuli of 6 V. Simultaneously, the optical visibility is regulated between opaque and transparent states in response to alternate electric‐trigger. Upon newly explored in situ monitoring techniques in terms of optical microscopes and spectrums, the switching principle has been successfully uncovered. That is, in the presence of Joule‐heat, the melt paraffin suffering from swelling enables a slippery ALS system within 20 s to contribute light passage channel and low *F*_CAH_. Once the Joule‐heat is discharged, an undulating nonslippery ASS system would reconfigure within 8 s in terms of the cold‐shrinkage of solidified‐paraffin for rendering light blockage and high *F*_CAH_. Moreover, the quantitative relationship among serving voltage and surface temperature and response time, together with the on–off state transparency as a function of laser power, is systematically investigated. Thanks to its portable merit, in situ thermal management, programmable visibility, as well as steering diverse functionalized droplets by electric‐triggered JR‐PISSs are successfully deployed. Compared with the previous Nepenthes‐inspired slippery surfaces, current electric‐activated JR‐PISS is more competent for on‐demand regulating optical and wetting properties in synergy. This work provides deep insight for designing thermo‐involved slippery surfaces and further bloom the studies of microfluidics, anti‐icing, antifouling, and smart windows, and so on.

## 4 Experimental Section

### Materials

Highly‐transparent (>95%) elastomeric polydimethylsiloxane (PDMS) films (thickness: 200 *μ*m) were donated by Bald Advanced Materials Co., Ltd. AgNO_3_ (99.8%) was obtained from Shanghai Qiangshun Chemical Reagent Co., Ltd. Polyvinylpyrrolidone (PVP, MW of 55 000) and hydroxypropyl methylcellulose (HPMC) were harvested from Sigma‐Aldrich. Ethylene glycol (EG), glycerol, NaCl, NaBr, and acetone were purchased from Sinopharm Chemical Reagent Co., Ltd. Sago 9760 and Sago 3223 were obtained from Sago Co., Ltd. Silver paste was brought from Shenzhen Sinwe New Material Co., Ltd. Paraffin wax (*T*_m_ ≈ 52^o^) was provided by Jinan Dingyi Chemical Co., Ltd. Fe_3_O_4_ nanoparticles (diameter: 10 nm) were contributed by Tianjin kaili metallurgical research institute. PDMS silicone elastomer obtained from Dow Corning (Sylgard 184) was used as the bonding agent. PDMS is a two‐part solvent free flexible silicone organic polymer in the form of a base compound with a separate hydrosilane curing agent that acts as a crosslinker. Distilled water (H_2_O, 1 g cm^–3^ density) served as contact‐angle test materials.

### Femtosecond Laser Engineering

The superhydrophobic micropillar‐arrayed PDMS membrane (SPAM) was manufactured by one‐step cross‐scanning of femtosecond laser. The laser beam (104 fs, 1 kHz, 800 nm) derived from a regenerative amplified Ti:sapphire femtosecond laser system (Legend Elite‐1K‐HE, Coherent) was carried out for ablation. During the fabrication process, the laser beam was guided onto the platform via a galvanometric scanning system (SCANLAB), which enabled the laser beam focus and scan along the *x* and *y* coordinate directions. The laser pulse number, scan spacing, and speed were in sequence fixed at 50 µm, 100 µm, and 5 mm s^−1^, respectively. Thereby, a series of super‐hydrophobic SPAMs could be resultant from programmable laser with laser powers of 0.05, 0.1, 0.2, and 0.3 W, respectively.

### Building of Smart JR‐PISS—Synthesis of High‐Quality SNWs

For synthesis, three precursor solutions of A) 0.220 M NaBr, B) 0.210 M NaCl, and C) 0.505 M PVP in EG were individually prepared for utilization. Fresh AgNO_3_ was dissolved in EG under the assistance of ice‐cold ultrasonic bath (4–8 °C) for 5 min. Thereafter, 2.5 mL of A, 5 mL of B, 25 mL of C, and 25 mL of AgNO_3_ (0.265 m) were successively added to a 100 mL flask placed in an oil bath at room temperature. Vigorous stirring was applied for 30 min, and then the temperature in the flask was elevated to 170 °C in 15 min, where the nitrogen gas with a flux of 150 mL min^–1^ was bubbled through the reaction. Subsequently, the flask was corked, and the reaction was left for 1 h without disturbing. The flask was then taken off from the oil bath immediately and transferred to the cold water bath (≈25^o^) for cooling once the reaction was terminated.^[^ ^20^ ^]^

### Building of Smart JR‐PISS—Formulation of SNW Ink

A total of 16 mg of purified SNWs harvested by a positive‐pressure filtration and acetone purification procedure were dissolved in 16 mL of DI water with the assistance of 32 mg of HPMC and a Sago‐dispersant (v/v 0.0025%), and a Sago‐flatting agent (v/v 0.0025%). Finally, 1 mg mL^–1^ SNW ink could be obtained after the mixture was fixed on a table concentrator and allowed to blend for 1.5 h with a rotating speed of 110 rpm.^[^ ^20a^ ^]^

### Building of Smart JR‐PISS—Preparation of Transparent SNWH

An automatic coating machine (BEVS 1811/2) equipped with an OSP‐40 scraper was utilized to coat the conductive SNW network on flexible PET substrate, where the coating rate and area were fixed at 120 mm s^–1^ and A4 (21.0 cm × 29.7 cm), respectively. The starting button was pressed after the dropping of 1 mL of SNW ink, and highly conductive SNW films were obtained after a brief annealing process at 60 °C for 5 min. Thereafter, two symmetric copper‐wire electrodes could be soldered using patterned oblong silver paste. SNWH could be prepared after annealing at 70 °C for 5 h, which was then bonded to SPAM depending on a thin‐layer PDMS suffering from successive bubble removal (vacuum oven, 30 min), spin‐coating (room temperature, 2500 rpm, 40 s), and curing (80 °C, 2 h).

At a final stage, a kind of thermal‐spin‐coating (TSC) method in our previously explored work was employed for lubricating the solid paraffin wax into the as‐prepared SPAM. Typically, SPAM with an embedded SNWH was fixed on a glass slide by 3M adhesive tape, which was then immobilized on the platform of spin‐coater by virtue of vacuum pump. Afterward, a patch of solid paraffin wax (≈23 mg) was released on SPAM and allowed to melt and spread for 2 min under the assistance of a top infrared radiation (IR) lamp, where the distance between the IR lamp and SPAM were fixed at ≈10 cm. Meanwhile, a spin‐coating program (500 rpm, 30 s) was initiated for uniformly lubricating the liquefied paraffin into SPAM. Finally, a sandwich‐structured JR‐PISS could be readily harvested after a brief condensation process at room temperature.

### Characterization

Morphology of silver nanowire and micro/nanostructures induced by laser ablation for SPAMs were characterized by using a field‐emission scanning electron microscope (JSM‐6700F). The sliding angles of the water, NaCl solution, glycerol, and EG droplets were measured using a CA100C contact‐angle system (Innuo) at 10% humidity and 20 °C. The sheet resistance of SNW films was measured using a four‐point probe technique (RST‐9, Four‐Probe Technology). The surface temperatures of the SNWH and JR‐PISS were measured by a thermal infrared camera (VarioCAMhr head 680, InfraTec). Optical transparency evolution curves for JR‐PISSs were recorded by dynamic‐spectrum‐analysis mode of UV‐2501PC/2550 (Shimadzu Corporation, Japan). The near‐infrared light (FU808AD300‐BC/BD10, Fuzhe Technology Co., Ltd, China) with wavelength of 808 nm (0.3 W; spot area, 1.4×2.3 mm^2^) was used for the proof‐of‐concept of JR‐PISS serving as light shutter. All digital photos were shot by a mobile phone (iphone‐8 plus, 7 mega‐pixel). 3D images for in situ monitoring the profile evolution of JR‐PISS with respect to Joule‐heating times were captured by a 3D profile meter (VK‐X100, KEYENCE CORPORATION, Japan).

## Conflict of Interest

The authors declare no conflict of interest.

## Supporting information

Supporting Information

Supplemental Movie 1

Supplemental Movie 2

Supplemental Movie 3

Supplemental Movie 4

Supplemental Movie 5

Supplemental Movie 6

Supplemental Movie 7

Supplemental Movie 8

Supplemental Movie 9

## Data Availability

Research data are not shared.
