# Supplementary material for: In Situ Electric‐Induced Switchable Transparency and Wettability on Laser‐Ablated Bioinspired Paraffin‐Impregnated Slippery Surfaces
Source: Adv Sci (Weinh). 2021 May 29;8(14):2100701. doi: 10.1002/advs.202100701 (PMC8292917; doi:10.1002/advs.202100701)
Supplement: Supplementary file 1 — Supporting Information [file ADVS-8-2100701-s008.pdf]

© 2021 Wiley-VCH GmbH

ADVANCED SCIENCE
Open Access

Supporting Information

for *Adv. Sci.*, DOI: 10.1002/advs.202100701

In Situ Electric-induced Switchable Transparency and Wettability on Laser-Ablated Bioinspired Paraffin-impregnated Slippery Surfaces

Chao Chen, Zhouchen Huang, Suwan Zhu, Bingrui Liu, Jiawen Li, Yanlei Hu, Dong Wu* and Jiaru Chu*

In Situ Electric-induced Switchable Transparency and Wettability on Laser-Ablated Bioinspired Paraffin-impregnated Slippery Surfaces

Chao Chen, Zhouchen Huang, Suwan Zhu, Bingrui Liu, Jiawen Li, Yanlei Hu, Dong Wu* and Jiaru Chu*

C. Chen, Z. Huang, S. Zhu, B. Liu, Prof. J. Li, Prof. Y. Hu, Prof. J. Chu, Prof. D. Wu,
CAS Key Laboratory of Mechanical Behavior and Design of Materials, Department of Precision Machinery and Precision Instrumentation, University of Science and Technology of China, Hefei 230026, China
E-mail: jwl@ustc.edu.cn and dongwu@ustc.edu.cn

Keywords: femtosecond laser, bioinspired electric-triggered slippery surfaces, transparent silver nanowire thin-film heater, in situ controllable wettability, switchable visibility

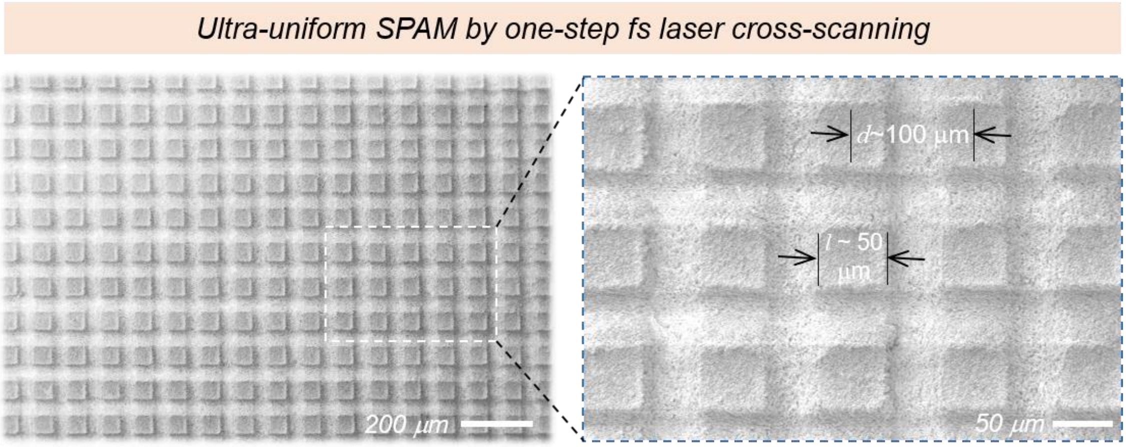

Figure S1. SEM clips for typical SPAM resulted from one-step fs laser ablation with power of 0.20 W. The left verifies the good uniformity of SPAM by current laser processing method and the right displays that there are indeed a variety of micro- and nano-structures accompanying the pillars of SPAM, which is convinced to be conducive for the lubrication and storage of paraffin wax.

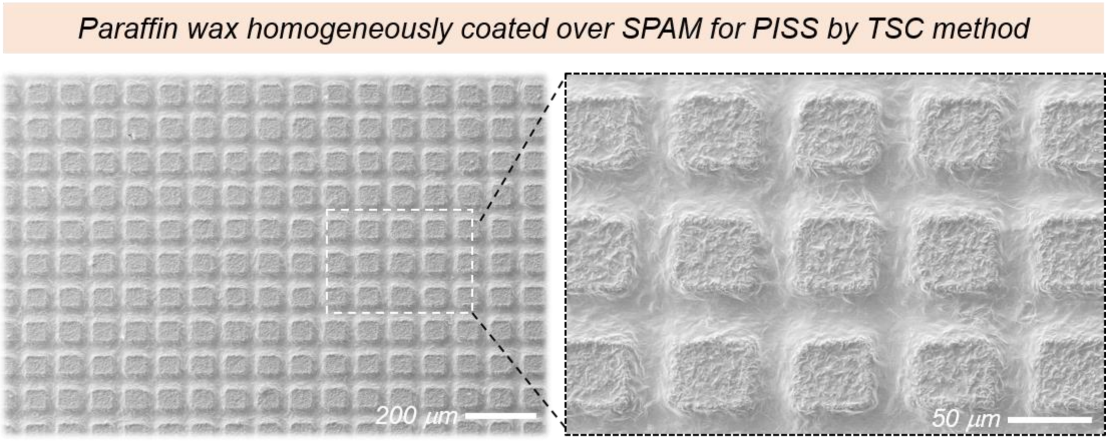

Figure S2. SEM images for typical PISS by lubricating paraffin wax (thermal-spin-coating method-1500 rpm-60 s) into the as-prepared SPAM (0.2 W). The results show that the paraffin wax could be homogeneously impregnated into the resultant SPAM, though a great number of wrinkles arising from cold-shrinkage had been observed

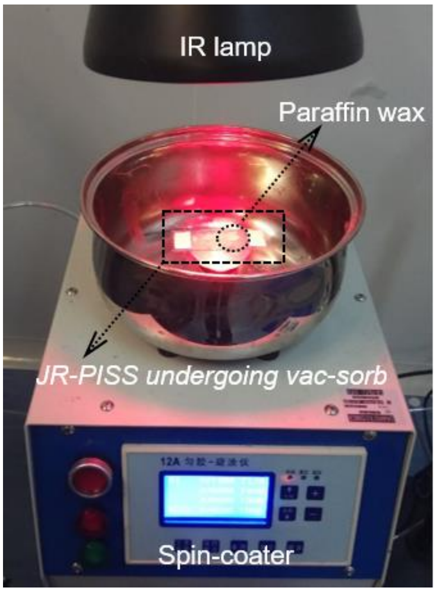

Figure S3. Digital picture for a home-built thermal-spin-coating (TSC) facility composed of a top IR lamp and a spin-coater equipped with a *vac-sorb* pump. Under the assistance of IR lamp heating, a piece of solid paraffin wax can uniformly melt and lubricated into the as-prepared SPAM for JR-PISS after removing IR lamp and a cold-shrinkage process.

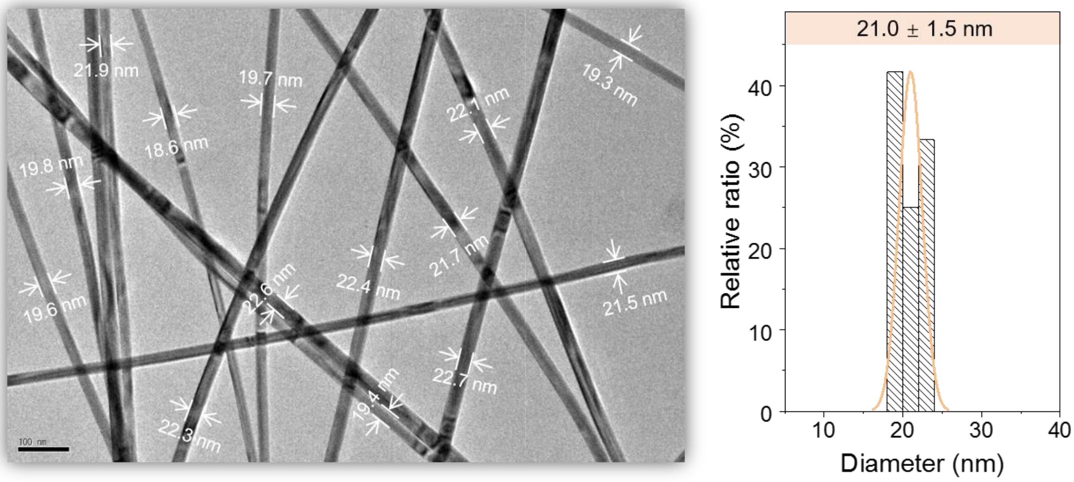

Figure S4. TEM image and the statistic Gaussian distribution for characterizing the diameter of as-prepared silver nanowires.

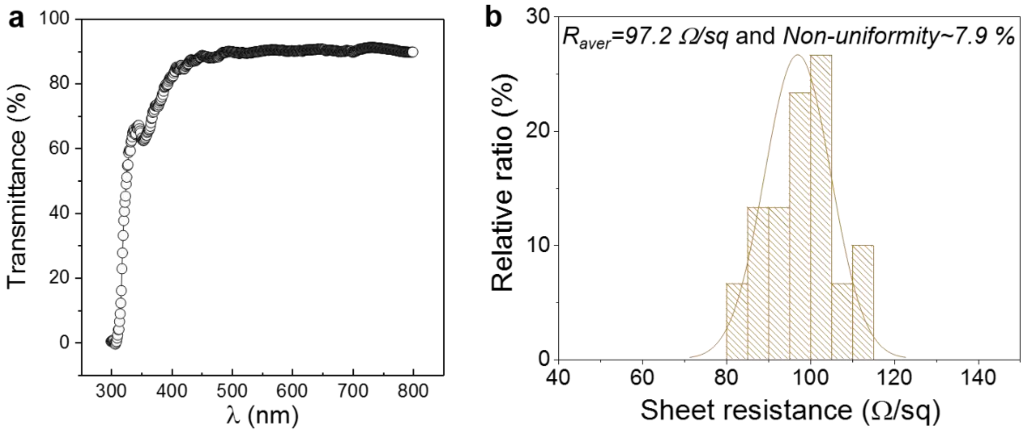

Figure S5. (a) UV-Vis spectrum and (b) numerical statistic of sheet resistance and its Gaussian distribution for the resultant transparent conductive silver nanowire thin-film. The results reveal that the underlying SNWH has an excellent transmittance (~90%) and low sheet resistance (97.2 Ω/sq), as well as homogeneous conductive network (NUF~7.9%).

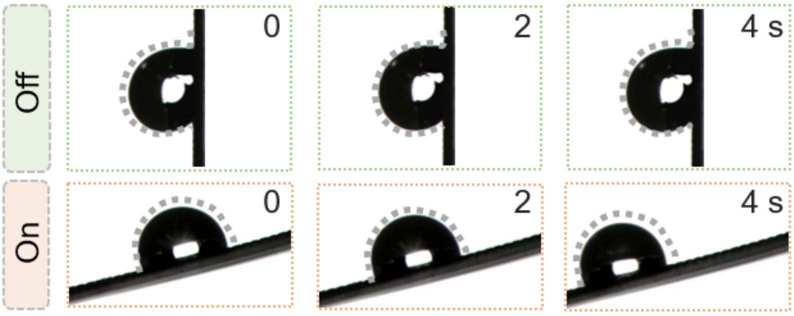

Figure S6. Dynamic characterization of water droplet (5 μL) on JR-PISS in response to electric trigger (6 V). The upper line represents the case of pinning a drop on its surface at the absence of Joule-heat on account of its exaggerated adhesion force arising from the solidified

paraffin; The lower line unfolds the case of slide a drop in the presence of Joule-heat because of low hysteresis donated by liquefied paraffin.

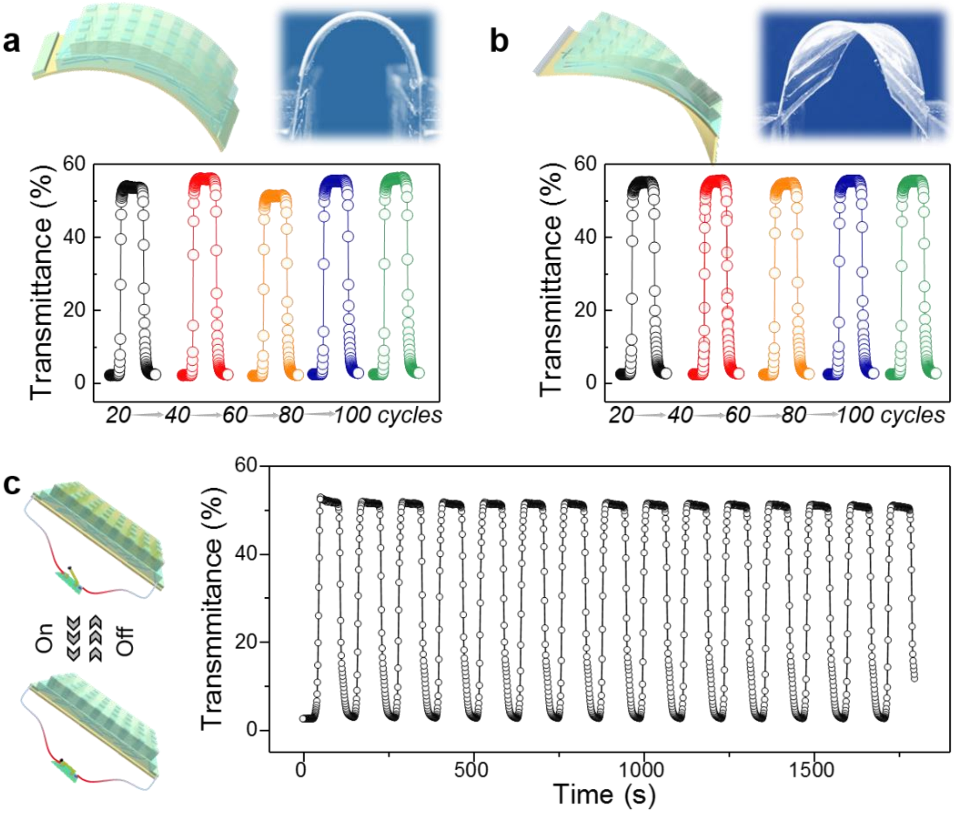

Figure S7. Longevity test for JR-PISS in cases of cyclic (a) bending, (b) twisting and (c) heating-freezing. The results demonstrate that current electric-actuated JR-PISS has good stability in terms of flexibility and reconfiguration.

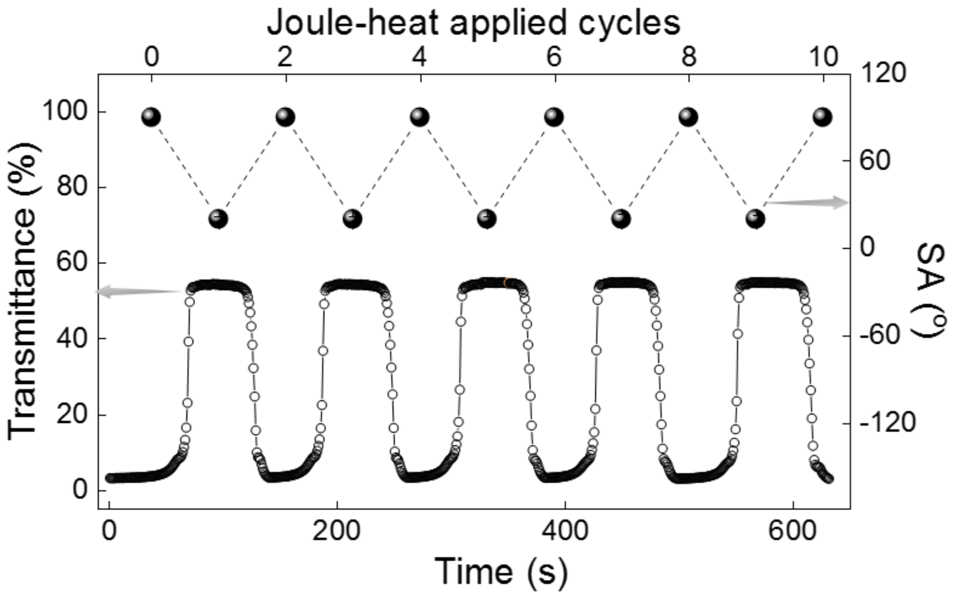

Figure S8. Cyclic longevity test of optical visibility and wettability for JR-PISS in response to the alternate electric stimuli. The result shows that current intelligent device, that is, JR-PISS has a good reconfiguration ability for switching the optical and wetting performance.

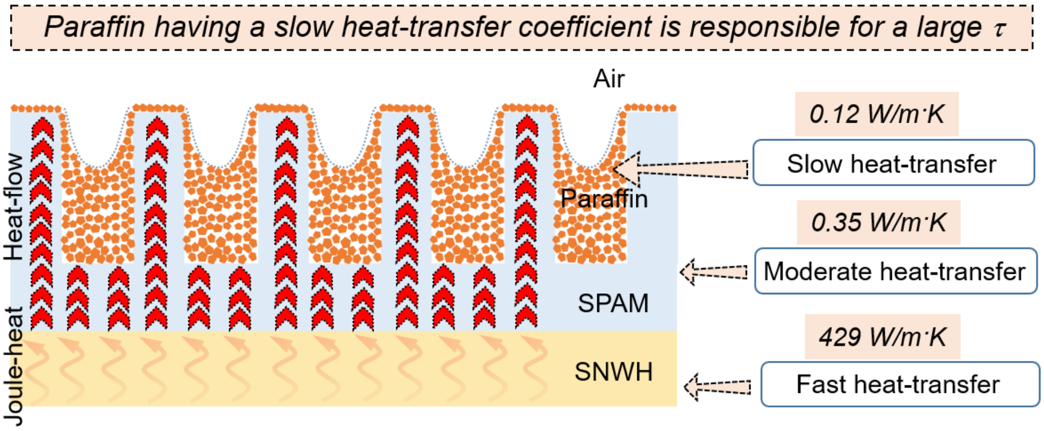

Figure S9. Schematic diagram for presenting the thermodynamics, that is, heat-transfer process among media of underlying SNWH, in-between SPAM and terminal infused paraffin wax. The result manifests that the long response time of JR-PISS than that of SNWH should be ascribed to the far smaller thermal-conductivity (0.12 W/m·K) of paraffin in comparison with that of silver nanowire (429 W/m·K), which should be responsible for the three processes including solid paraffin adjacent to SNWH melt preferentially, liquid paraffin swelling to elevate the lubricant layer and accomplishment of liquidation for a smooth terrain.

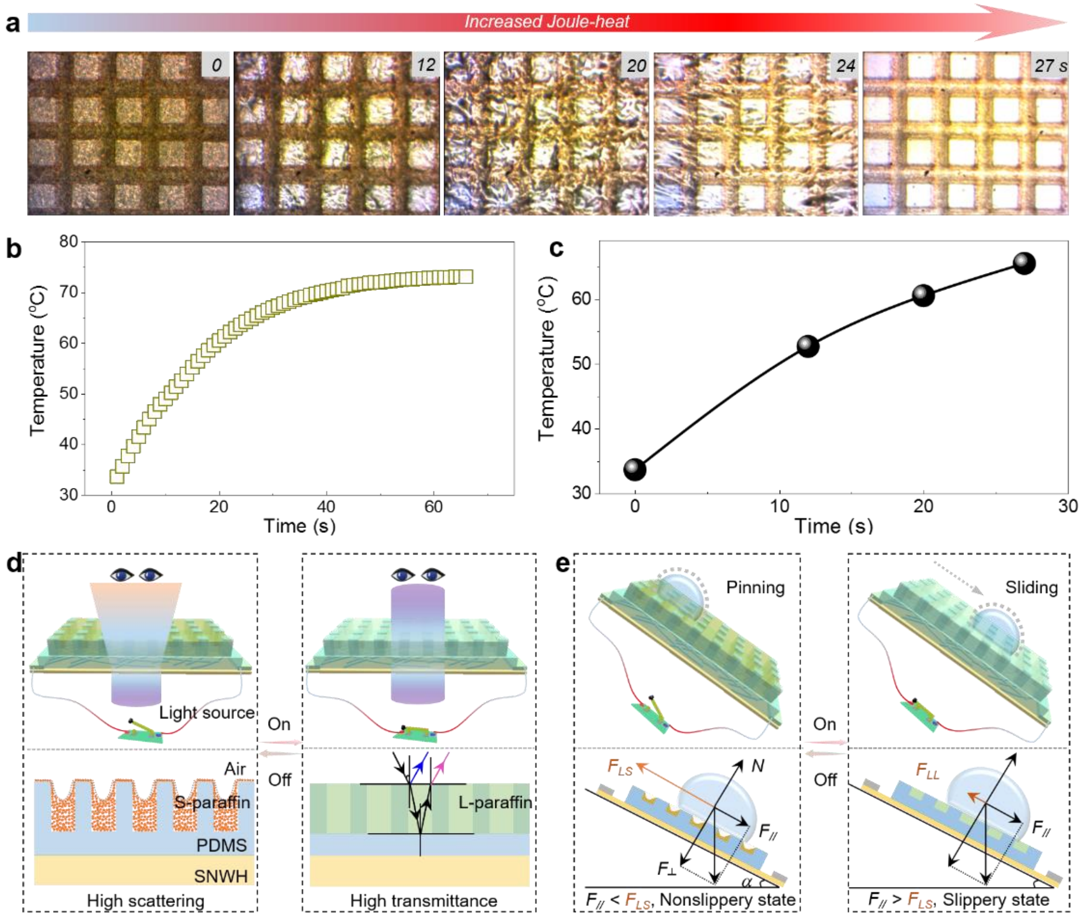

Figure S10. (a) In situ monitoring the morphology evolution of paraffin infused in SPAM with the Joule-heating time in a top view. The result uncovers that the infused solidified paraffin undergoes three crucial thermodynamic processes including melt, swelling, and liquefied. (b) Temperature-time curve for JR-PISS that was loaded with a low voltage of 6 V. (c) Temperature change according to time obtained from (b). The result shows that the slow heat transfer process should be responsible for the lagging of paraffin's conversion between

solidified and liquefied states. Schematic diagram for illustrating (d) the optical and (e) the wetting performance in response to Joule-heat. The results reveal that JR-PISS tends to hinder the passage of incident light and impede the sliding of surface droplets without Joule-heat because of the higher scattering and exaggerated hysteresis resulted from the solidified paraffin.

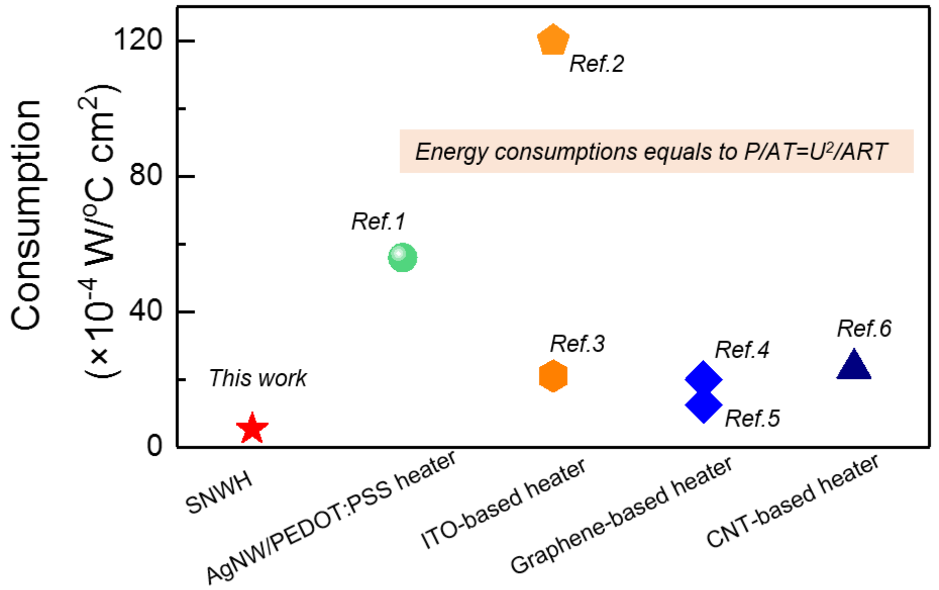

Figure S11. Comparisons of energy consumption for currently widely-used heaters including heaters woven by conductive carbon nanotube (CNT), graphene, indium tin oxide (ITO), hybrid of silver nanowire and PEDOT:PSS, and pure silver nanowire in this work. Obviously, pure silver nanowire heater (SNWH) unfolds the most energy-saving merit because of its lower conductivity and smaller junction resistance.

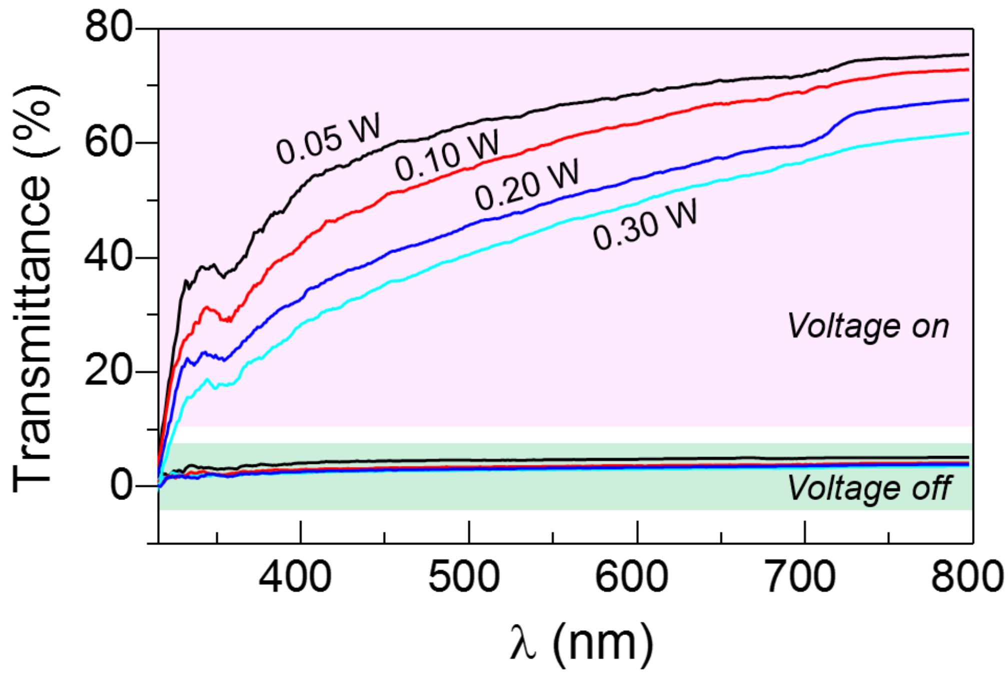

Figure S12. UV-Vis spectrum for four different JR-PISSs in response to electric trigger (6 V) integrated by SPAMs that were fabricated by fs laser powers of 0.05, 0.1, 0.2 and 0.3 W, where the thermal-spin-coating speed and time were fixed at 1500 rpm and 60 s. The larger the fs laser ablating power, the smaller the optical transmittance of JR-PISS.

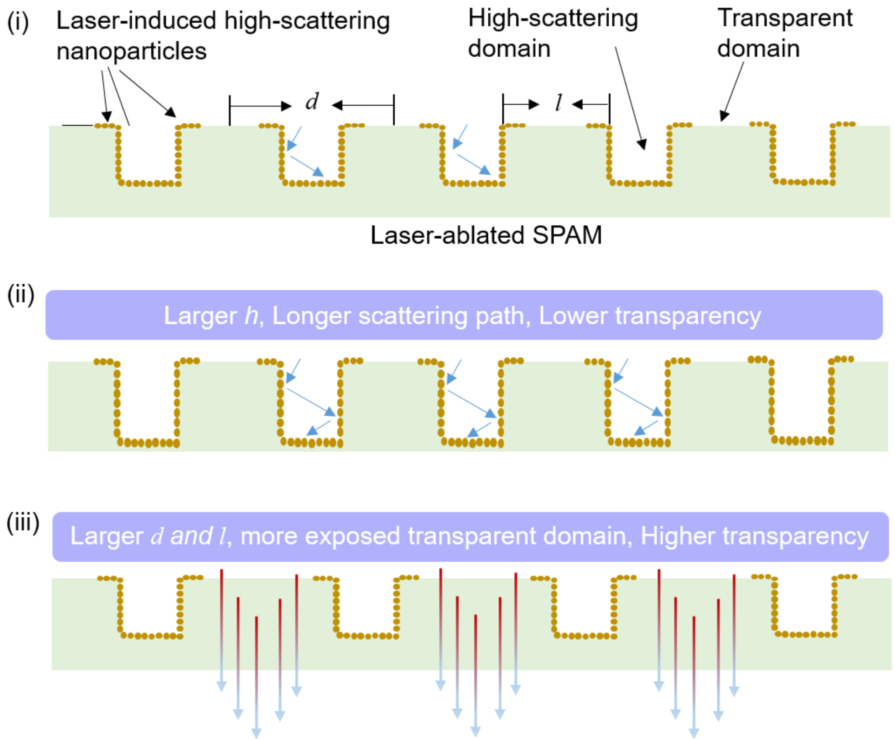

Figure S13. Schematic diagram for unfolding the effect of pillar width (*l*), distance (*d*), together with height (*h*) on the performance of laser-ablated SPAM and resultant JR-PISS. Indicating that the designed SPMA with larger *d* and *l* and smaller *h* should be favourable for further improving the performance of JR-PISS in the future work.

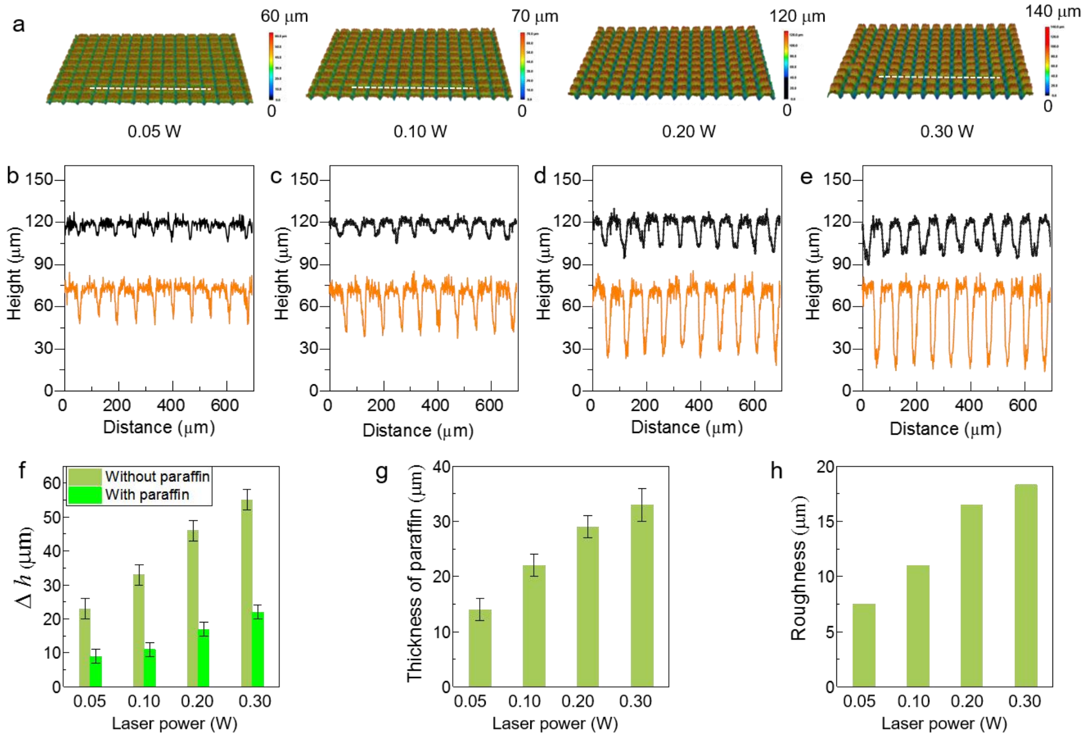

Figure S14. (a) 3d images for SPAMs ablated by fs laser with power of 0.05, 0.1, 0.2 and 0.3 W, respectively. (b-e) The corresponding profiles obtained by line-scanning method in (a). (f) The difference of height (*Δ h*) between the top of a pillar and the bottom of a groove for cases of SPAMs and PISSs that were fabricated by various fs laser power, where the thermal-spin-coating speed and time were fixed at 1500 rpm and 60 s. (g) The thickness of lubricant

paraffin wax changes with fs laser ablating power that were calculated according to (f). (h) *Ra* evolution of SPAMs as a function of fs laser power. The results unfold that the larger the fs laser power, the larger the surface roughness for SPAMs and the deeper the ablated grooves and so the thicker the lubricated paraffin wax.

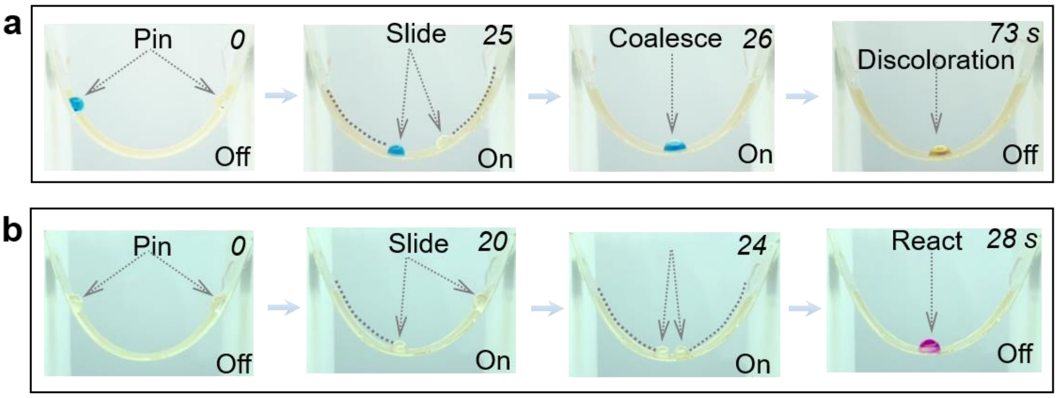

Figure S15. (a) Steering functionalized droplets (left: 5 μL, mixture of glucose and indigo carmine; right: 5 μL, NaOH aqueous solution) on curved JR-PISSs for their selective coalesce. (b) Actuating two colorless droplets (left: 5 μL, glycerol containing a small amount of phenolphthalein; right one: 5 μL, NaOH aqueous solution) toward the bottom of flexible JR-PISS successively for their in-situ reaction. The transporting speed for functionalized droplets could be adjusted by simply tuning the curvature of JR-PISS on account of its good flexibility.

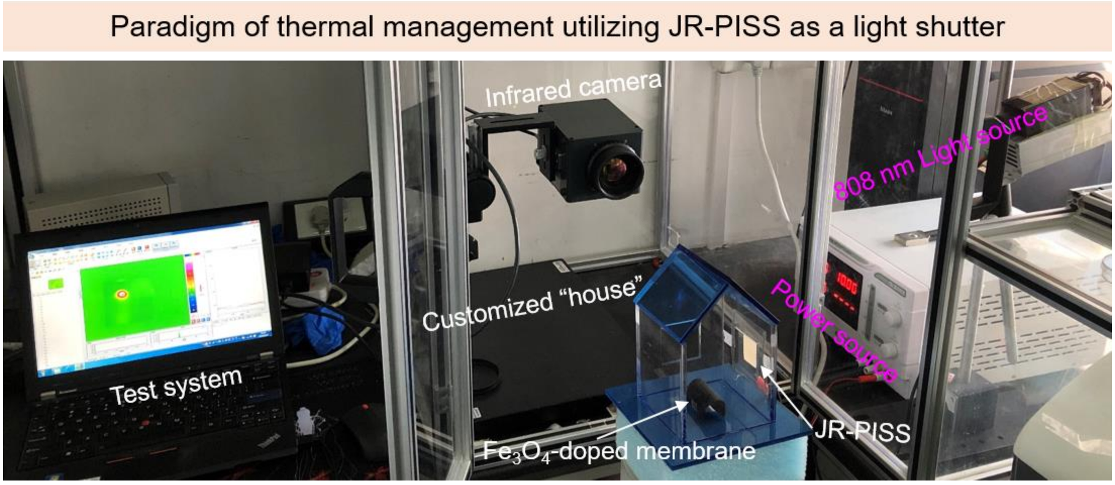

Figure S16. Digital picture for electric-triggered JR-PISS behaving as an optical shutter for in-situ temperature control. The home-made mimetic system contains a series of components including testing system, infrared camera, light source with a wavelength of 808 nm, DC power source, customized "house" equipped with JR-PISS and in-door Fe_3O_4 doped membrane (5 wt%). The 808 nm light would pass through JR-PISS to "warm" the indoor objective by photo-thermal effect because of the lower scattering of liquefied paraffin when the electric stimuli is switched on. In contrary, once the Joule-heat is in-situ discharged, the indoor target tends to "cool" depending on the strong scattering of solidified paraffin wax. Upon above method, current JR-PISS is envisioned to be applicative in smart windows that have bi-functional usages, that is, in situ switching optical visibility and surface wettability.

Table S1. Comparisons of structure, material and performance over stimuli-responsive SLIPS

	Nat. Mater. 2013, 12, 529	*Adv. Mater.* 2014, 26, 1895	*ACS Nano* 2016, 10, 9387	*ACS Appl. Mater. Interfaces* 2019, 11, 7591	*J. Mater. Chem. A*, 2019, 7, 18510	*Ind. Eng. Chem. Res.* 2019, 58, 6424	This work
Architecture	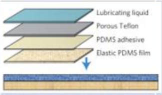	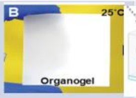	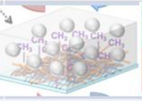	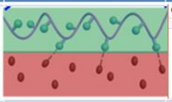	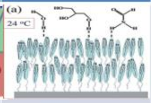	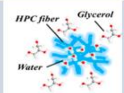	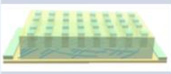
Components	Perfluoropolyether/porous Teflon/PDMS	Bulk PDMS/paraffin hybrid	CHINF&PAA /hybrid paraffin	ssDNA drop/lubricants/PMDS	Porous polystyrene (PS)/hybrid lubricants	HPC/glycerol	Parraffin/SPAM/SNWH
Type	Membrane	Membrane	Membrane	Membrane	Membrane	Membrane	**Intelligent electronic device**
Fabricating strategy	Bonding	Blending	LBL& Chemical hydrophobization	biochemical reaction	Spin coating/Freeze drying	Blending	Fs laser ablation/Automatic coating/Bonding
Stimuli	mechanical stretch	Temperature	Temperature	Temperature	Temperature	Temperature	**Electric**
Heating source	No	Ex-situ oven/hot-plate (220 V)	Room-temperature-reliable	Ex-situ oven/hot-plate (220 V)	Ex-situ oven/hot-plate (220 V)	Ex-situ oven/hot-plate (220 V)	**Joule heat (6V)**
Adaptive on 2D/3D surface	No	NA	NA	NA	NA	NA	**Yes**
Portability	Yes	No	No	No	No	Yes	Yes
Response time	NA	NA	NA	NA	NA	NA	**~20 s**
Functionality	Switching transparency and wettability	Switching transparency and wettability	Switching transparency and wettability	Switching wettability	Switching wettability	Switching transparency and wettability	Switching transparency and wettability
Tuning manner	Active	Active	Passive	Active	Active	Passive	Active

Table S2. Cost evaluation for constructing electric-induced JR-PISS

(1) Cost calculation over SPAM			
Fs Laser ablation (including laser, water-cooling and air conditioner systems, 6 kW)	Time-cost for processing 6×6 cm^2 SPAM (1.5 h)	Electric charge (1.5￥/kW·h)	Cost ①: 6 kW×1.5 h×1.5 ￥/kW·h = 13.5 ￥
**(2) Cost calculation over paraffin impregnation**			
Paraffin wax ~ 0.03g×0.809￥/g =0.024￥	Thermal-spin-coating period (60 s),power (0.07 kW)	Electric charge (1.5￥/kW·h)	Cost②:0.024￥ +0.07 kW×0.0166 h×1.5￥/kW·h = 0.0357￥
**(3) Cost calculation over silver nanowire coating and Bonding**			
Synthesize silver nanowires (16 mg): （Materials expenses） (NaCl) 0.0613 g×0.031￥/g = 0.002￥;(NaBr) 0.056 g×0.134￥/g = 0.0075￥;(PVP) 0.70 g×0.36￥/g = 0.252￥;($AgNO_3$) 2.1 g×2.6￥/g = 5.46￥; （Electric charge） Oil bath heating 1.25 h×2 kW×1.5￥/kW·h = 3.75￥			
Silver nanowires ink (16 mL, 1mg/mL): 32 mg HPMC+Sago-dispersant (v/v 0.0025%)+Sago-flatting agent (v/v 0.0025%) ≈ 0.06￥;			
Silver nanowire heater bonding (6×6 cm^2): PET film (6×6 cm^2) = 0.0005￥; PDMS adhesive layer ≈ 0.004￥ ; Silver paste for electrodes ~2 mg×0.013￥/mg=0.026￥ ; Here, the material expense for 1 mL SNW ink is 5.7815￥/16 mL ≈ 0.36￥/mL and thus the material cost for preparing 1 cm^2 silver nanowire film is calculated as 0.36￥divided by (21.0×29.7 cm^2) , that is, 0.0006￥/cm^2. Accordingly, the cost for silver nanowire heater with area of 6×6 cm^2 is 0.0216￥; Electric charge for oven heating 7h×0.6kW×1.5￥/kW·h=6.3￥			
**Total cost**			
13.5 ￥ (electric charge-SPAM)+0.0357￥+3.75￥ (electric charge-oil bath)+0.0005￥ (PET platform)+0.004￥ (PDMS adhesive layer for bonding)+0.0216￥ (material expense for silver nanowires film)+6.3￥ (electric charge for heating oven)+0.026￥ (silver paste) = 23.6378￥= 3.65 $			

Table S3. Thermodynamics involved key parameters for various components of JR-PISS including mass density (ρ), thickness (d), specific heat capacity (c) and their thermal conductivity (h).

Materials	ρ (g cm^{-3})	d (μm)	c (J/g °C)	h (W/m·K)
SNW	10.5	NA	0.233	429
PET	1.4	100	1.1-1.4	0.25
PDMS	NA	200	NA	0.35
Paraffin wax	0.9	29.1	2.6	0.12

Refferences

[1] S. Ji, W. He, K. Wang, Y. Ran, C. Ye, *Small* **2014**, *10*, 4951.

[2] J. Jensen, M. Hosel, I. Kim, J. Yu, J. Jo, F. C. Krebs, *Adv. Funct. Mater.* **2014**, *24*, 1228.

[3] S. H. Park, S. M. Lee, E. H. Ko, T. H. Kim, Y. C. Nah, S. J. Lee, J. H. Lee, H. K. Kim, *Sci. Rep.* **2016**, *6*, 33868.

[4] J. J. Bae, S. C. Lim, G. H. Han, J. Y. Wo, D. L. Doung, E. S. Kim, S. J. Chae, T. Q. Huy, N. V. Luan, Y. H. Lee, *Adv. Funct. Mater.* **2012**, *22*, 4819.

[5] D. Sui, Y. Huang, L. Huang, J. Liang, Y. Ma, Y. Chen, *Small* **2011**, *7*, 3186.

[6] J. Yan, Y. G. Jeong, *Mater. Design* **2015**, *86*, 72.
